# Imaging of Multiple Myeloma: Present and Future

**DOI:** 10.3390/jcm13010264

**Published:** 2024-01-02

**Authors:** Víctor Rodríguez-Laval, Blanca Lumbreras-Fernández, Beatriz Aguado-Bueno, Nieves Gómez-León

**Affiliations:** 1 Department of Radiology, University Hospital La Princesa, IIS-Princesa, Calle Diego de León 62, 28005 Madrid, Spain; blancalumfer@gmail.com (B.L.-F.); nievesgleon@gmail.com (N.G.-L.); 2 Department of Medicine, Autonomous University of Madrid, Calle del Arzobispo Morcillo 4, 28029 Madrid, Spain; 3 Department of Hematology, University Hospital La Princesa, IIS-Princesa, Calle Diego de León 62, 28005 Madrid, Spain; baguadobueno@gmail.com

**Keywords:** positron emission tomography, computed tomography, multiple myeloma, magnetic resonance imaging, imaging

## Abstract

Multiple myeloma (MM) is the second most common adult hematologic malignancy, and early intervention increases survival in asymptomatic high-risk patients. Imaging is crucial for the diagnosis and follow-up of MM, as the detection of bone and bone marrow lesions often dictates the decision to start treatment. Low-dose whole-body computed tomography (CT) is the modality of choice for the initial assessment, and dual-energy CT is a developing technique with the potential for detecting non-lytic marrow infiltration and evaluating the response to treatment. Magnetic resonance imaging (MRI) is more sensitive and specific than 18F-fluorodeoxyglucose positron emission tomography/computed tomography (FDG-PET/CT) for the detection of small focal lesions and diffuse marrow infiltration. However, FDG-PET/CT is recommended as the modality of choice for follow-up. Recently, diffusion-weighted MRI has become a new technique for the quantitative assessment of disease burden and therapy response. Although not widespread, we address current proposals for structured reporting to promote standardization and diminish variations. This review provides an up-to-date overview of MM imaging, indications, advantages, limitations, and recommended reporting of each technique. We also cover the main differential diagnosis and pitfalls and discuss the ongoing controversies and future directions, such as PET-MRI and artificial intelligence.

## 1. Introduction

Multiple myeloma (MM) is the second most common adult hematologic malignancy, accounting for 10% of all hematological cancers, with an incidence of about 4/100,000/year and a median age at diagnosis of 70 years. The diagnosis of MM is based on a wide range of features, including biological findings, i.e., hypercalcemia, renal failure, and anemia, as well as the detection of lytic bone lesions on imaging studies (“CRAB criteria”) and pathological demonstration of bone marrow infiltration by monoclonal plasma cells [1]. It is almost always preceded by a premalignant stage known as monoclonal gammopathy of undetermined significance (MGUS). Possible additional intermediate stages include solitary plasmacytoma and smoldering multiple myeloma (SMM). Imaging plays a crucial role because the presence of bone lesions is one of the criteria for diagnosing MM.

International guidelines currently recommend low-dose whole-body computed tomography (LDWBCT), magnetic resonance imaging (MRI) covering the axial skeleton or the whole-body (WBMRI), and FDG-PET/CT as the imaging methods in the presence of a suspected diagnosis of MM [2,3]. A skeletal survey was the previous gold standard in MM, but it has limited sensitivity to detect lytic lesions [4]. In addition, it is not possible to assess extramedullary disease. The severity of the disease may be underestimated if the sole presence of osteolytic lesions is considered.

Whole-body techniques have greater sensitivity and can also evaluate the marrow composition. This is crucial because bone involvement is often heterogeneous both genomically and in its distribution and degree of infiltration within a patient [5,6,7]. WBMRI and FDG-PET/CT enable the demonstration of tumor infiltration before significant bone destruction occurs [8]. Early diagnosis is essential because early intervention improves survival in asymptomatic high-risk patients. Therefore, both patients with and without [4,5,6,7] symptoms can benefit from the prognostic value of FDG-PET/CT and MRI findings.

In this paper, we review the updated recommendations and recent developments of whole-body modalities, focusing on advanced imaging tools. The remaining relevant challenges and controversies are also addressed.

## 2. Imaging Techniques and Algorithm

**Figure 1:**
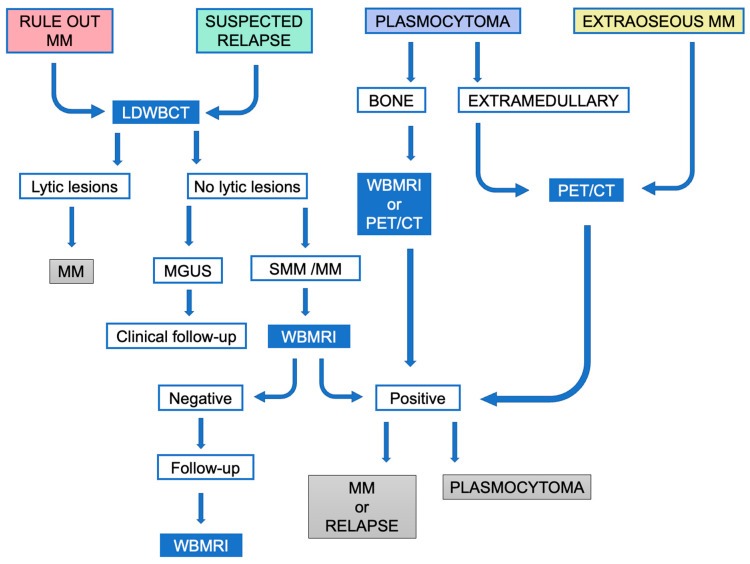
Simplified algorithm for the diagnostic imaging management spectrum of monoclonal plasma cell disorders according to the IMWG consensus recommendations.MM: Multiple myeloma; SMM: smoldering multiple myeloma.

The International Myeloma Working Group (IMWG) provides guidelines on the optimal use of imaging methods at different disease stages to standardize imaging for monoclonal plasma cell disorders worldwide [9] (Figure 1). Furthermore, we describe each technique’s advantages, as well as its disadvantages (Table 1).

**Figure 2:**
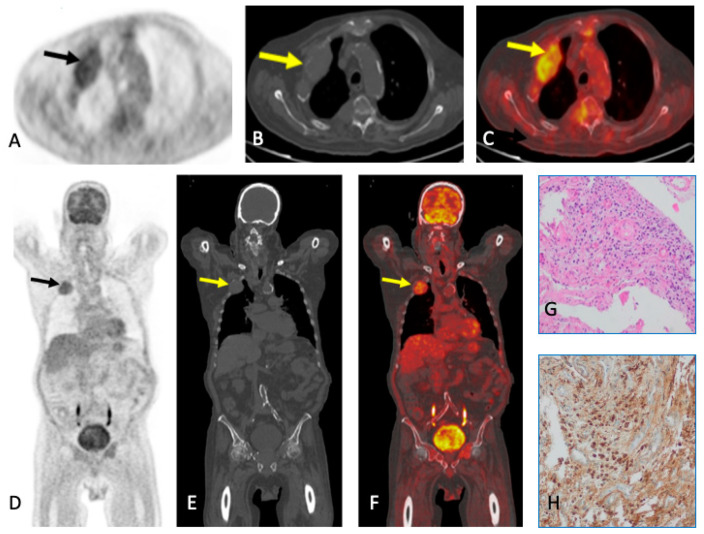
A 76-year-old man with IgG light chains in serum. Lytic lesion with soft tissue mass in the second right anterior costal arch with avid radiotracer uptake (arrows) in FDG-PET/CT in axial (top row) and coronal (bottom row) showing 18FDG uptake (**A**,**D**), CT (**B**,**E**), and fused images (**C**,**F**). Biopsy samples (**G**,**H**): the histological study revealed plasma cell proliferation leading to the diagnosis of MM.

**Figure 3:**
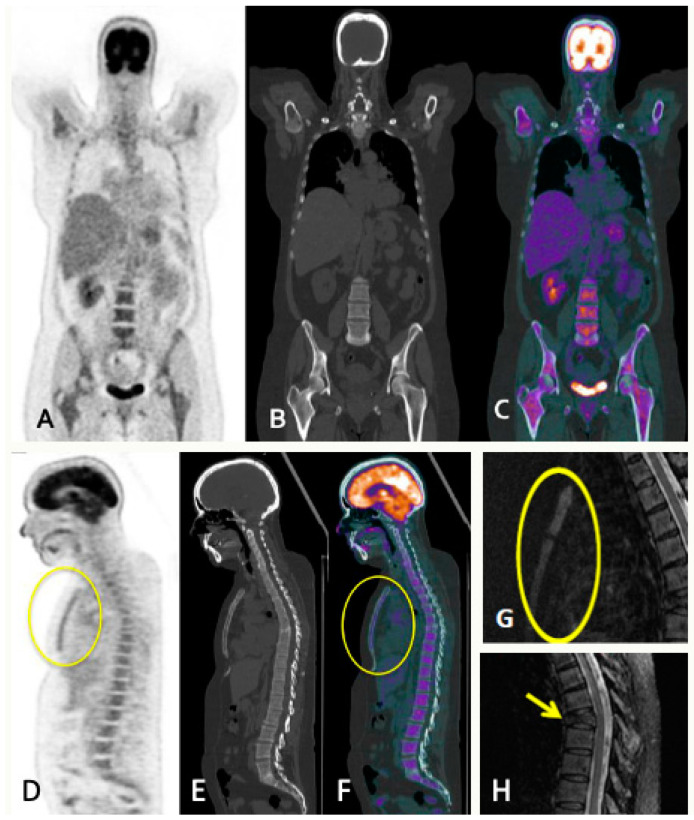
A 56-year-old female with SMM presenting lumbar pain resistant to analgesia. PET/CT images in coronal (**A**–**C**) and sagittal (**D**–**F**) show diffuse MM involvement of the bone marrow with intense metabolic activity in the axial and peripheral skeleton, and sternal involvement (circles). Spine STIR sagittal sequence (**G**,**H**) showing increased bone marrow sternal signal (circle), concordant with FDG-PET/CT findings and burst fracture in T7 vertebral body with slight posterior wall retropulsion (arrows) poorly visualized in the FDG-PET/CT study because of its lower resolution and absence of metabolic activity.

LDWBCT is necessary to rule out lytic lesions in suspected high-risk MGUS, SMM, and MM to increase sensitivity/accuracy in the diagnosis of MM. PET/CT or LDWBCT can be used for both baseline assessment and response, depending on availability. If LDWBCT is positive in MGUS, FDG-PET/CT must be performed. If negative, unless there are clinical signs of progression, no imaging tests are needed. In suspected SMM and MM with negative LDWBCT, WBMRI is recommended. FDG-PET/CT can be used in place of LDWBCT and WBMRI (Figure 2 and Figure 3). The same technique must be used for follow-up to provide comparability; in the case of SMM, this is necessary for the first five years. 

In clinical trials, FDG-PET/CT is the preferred imaging method to assess response. Residual lesions at FDG-PET/CT mean a high risk of an early progression whereby yearly follow-up is advisable. In the presence of an unequivocal lesion in a WBMRI, a six-month follow-up with alternate LDWBCT and WBMRI scans is advised. An increase in the number or size of focal lesions in MRI implies progression [9].

In the presence of bone plasmacytoma, WBMRI is recommended, as diffuse infiltration should lead to further examinations to rule out MM. If not available, FDG-PET/CT is an alternative. In extramedullary lesions, FDG-PET/CT is the preferred modality. The same initial exam should be repeated annually throughout the initial five years of follow-up. If the initial FDG-PET/CT was negative or not performed, LDWBCT is recommended as a baseline reference [9]. 

In suspected relapse, LDWBCT must be performed to assess bone destruction. A comparison should be made with the exam after therapy instead of with the initial one to avoid overestimating the extent of bone disease. Other malignancies should be ruled out, and, when in doubt, samples must be obtained for histological study, especially in the case of plasmacytoma or extraosseous disease. 

The real-world implementation of the IMWG recommendations is affected by a variety of factors, even though they serve as a model for imaging in MM. Institutions have different levels of access to imaging resources and techniques. Financial restrictions on patients can also be considered a limitation. Furthermore, patient characteristics such as claustrophobia or incompatible hardware may affect the choice of technique. MRI is the modality of choice when imaging is required to determine whether a vertebral fracture is benign or malignant or to assess spinal cord compressions [10]. As a result, while efforts to follow current guidelines are justified, flexibility is also required to take into account the system boundaries of each practice.

## 3. WBLDCT

WBLDCT has replaced the skeletal X-ray survey as the initial imaging technique of choice in monoclonal plasma cell disorders, according to the latest recommendations of the principal international societies’ guidelines (IMWG, European Society of Medical Oncology, and European Myeloma Network) [9,11,12]. For instance, a lesion cannot be seen on an X-ray unless there is up to a 50–75% loss of cancellous bone [4]. CT is also the imaging modality of choice as a guide for percutaneous biopsy and radiotherapy (RT) treatment planning [11,13].

### 3.1. CT Acquisition Protocol

WBLDCT is an imaging technique available in almost all specialized centers. Image acquisition is fast, preparation is not required, and the scan must be performed from the cranial vault to at least the proximal tibial metaphysis, including humeri. The radiation dose received with modern devices and low-dose protocols (100–120 kV) has been considerably reduced compared to conventional CT [14,15]. 

### 3.2. Imaging Findings

Typical imaging findings in untreated patients are lytic bone lesions greater than 5 mm (by convention to avoid a large number of false positives) with non-sclerotic rim, endosteal scalloping, cortical disruption, pathological fractures, and extraosseous soft tissue masses [14,15]. Lytic bone lesions are easily identifiable with no need for intravenous contrast, which is important in patients with decreased renal function secondary to the disease itself or additional pathologies [13,14]. 

**Figure 4:**
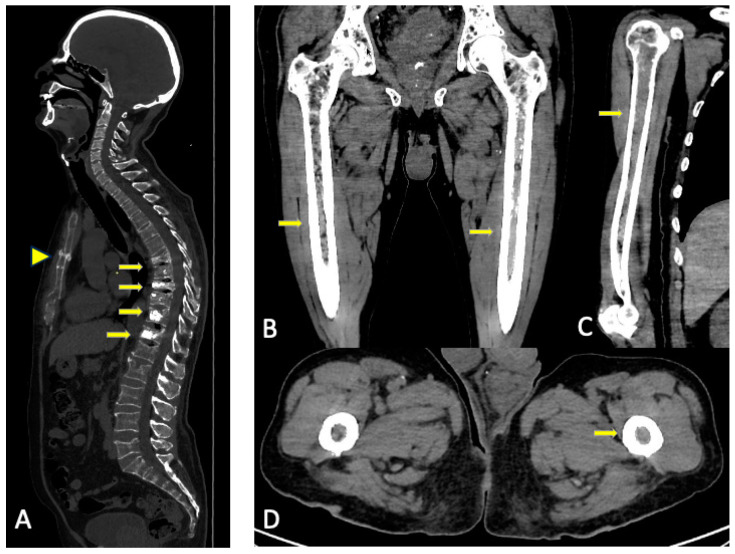
Conventional CT multiplanar reconstructions. (**A**) Reconstruction in the sagittal plane for better assessment of the spine and the sternum. Multiple fractures in the thoracic and lumbar spine, some of them with vertebroplasty (arrows) with no visible lytic lesions. Horizontal fracture in the sternal body (arrowhead). (**B**–**D**) Reconstruction in different planes of the long bones for better assessment of cortical and the medullar cavity. Substitution of fatty bone marrow for areas of soft tissue density without endosteal scalloping in both femora and right humeri (arrows). The asymmetrical distribution favors tumoral infiltration over red marrow reconversion.

Bone marrow infiltration without osteolysis is more easily detectable in long bones and manifests as pseudonodular lesions or areas of soft tissue attenuation (Figure 4). This finding in CT is not a defining event of MM but must raise suspicion and lead to further investigation. Long bone involvement generally entails a higher tumor burden, advanced disease, and worse prognosis [14,15]. 

### 3.3. Role of DECT

In addition to conventional monoenergetic CT, dual-energy CT (DECT) is gaining ground in this field. Conventional monoenergetic CT generates a photon beam centered around a single energetic peak, and the differentiation of structures is based on their different attenuation coefficient in the generated images. DECT technology differs in the acquisition of images at different energies, either by generating two beams with high and low energy coming from two X-ray tubes (dual-energy spectral CT) or using two detectors for high- and low-energy photons (dual-layer spectral CT).

Materials with different elemental compositions may have similar attenuation coefficients in monoenergetic TC images. DECT provides additional information through reconstruction algorithms that allows for the determination of material decomposition (calcium, fat, water, iodine, uric acid, etc.) based on the different interactions (different proportions of Compton and photoelectric effect) of each material with the spectrum of photon energies determined by their atomic weights and electron densities [16].

**Figure 5:**
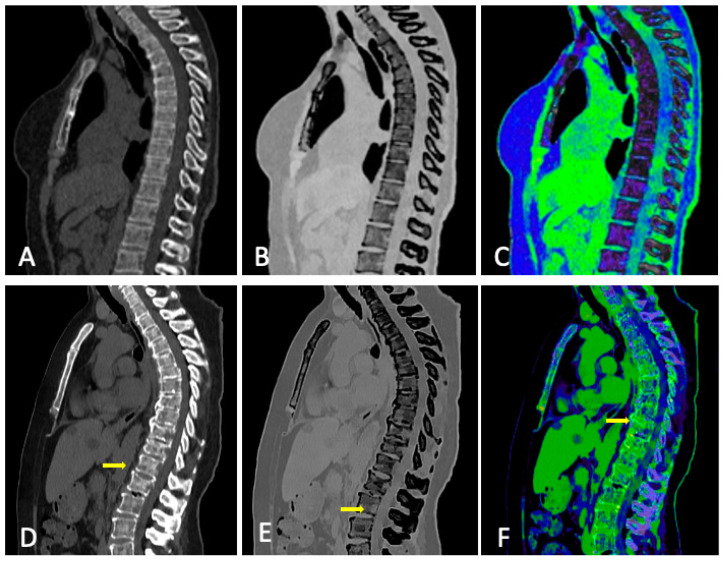
Normal bone (top row) vs. diffuse marrow infiltration with multiple osteolytic lesions (bottom row, arrows) in CT: (**A**,**D**) conventional CT, (**B**,**E**) grayscale VNCa image, and (**C**,**F**) color-coded VNCa image.

**Figure 6:**
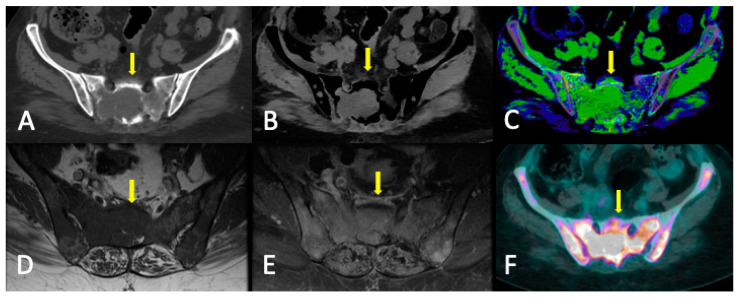
Sacral lytic lesion (yellow arrows) and diffuse marrow infiltration in the pelvis as the debut of MM with good correlation among conventional CT, DECT, MRI, and FDG-PET/CT. (**A**) Conventional CT. ROI average, 46 HU. (**B**) DECT VNCa grayscale (CaSupp index 25). ROI average, 3.2 HU. (**C**) DECT VNCa (CaSupp index 25) color-coded scale. (**D**) T1WI. (**E**) FST1WI after intravenous gadolinium administration. (**F**) FDG-PET/CT. Suvmax 29.5.

In the study of monoclonal plasma cell disorders, DECT enables a better assessment of bone marrow by creating postprocessed images with software, using the three-material decomposition: calcium, water, and fat. Calcium-suppressed or virtual non-calcium (VNCa) images allow for the differentiation of the fatty component of the bone marrow from other “watery” components, meaning hematopoietic bone marrow or tumoral cells. Some software allows for the adjustment of the degree of calcium suppression in the postprocess, changing the calcium suppression index (CaSupp). DECT also provides reconstructions of weighted average images similar to conventional monoenergetic CT images. This technique allows not only for the detection of lytic lesions and soft tissue masses, as with conventional monoenergetic CT, but also a qualitative and quantitative assessment of bone marrow composition [17,18]. Once the VNCa maps have been obtained, the software allows for the measurement of both the attenuation of the medullary component of regions of interest (ROI), whether they are lytic lesions or non-lytic soft tissue attenuation areas, as well as the creation of grayscale and color-scale maps for composition visual assessment. VNCa maps make it possible to study focal lesions, eliminating the imperceptible osseous component in the visual assessment of conventional CT images that increases the attenuation of the lesion in the quantitative study [19] (Figure 5 and Figure 6).

### 3.4. DECT vs. Conventional CT

Conventional WBLDCT can detect lytic lesions with only loss of 5% of trabecular bone [15]. With MRI as the reference standard, it has a sensitivity and specificity for the detection of lytic lesions that are 69.6% and 90.9%, respectively, greater than a skeletal survey, particularly in the spine and pelvis. It has a high positive predictive value (PPV) of 94.1%, but a modest negative predictive value (NPV) (58.8%), in the absence of lytic bone lesions [14,15]. VNCa color-coded images proved to be much more sensitive in the study of Kosmala et al. [20] in 2018 (91.3%) for the detection of bone involvement (including non-lytic infiltration) with the same specificity. Regarding the quantitative study of lytic lesions, Gu et al. [21] found that the mean subtracted value of the whole-skeleton calcium was lower than the mean of focal lesions values (−11.8 HU vs. −56.8 HU).

A recent study published in 2022 by Werner et al. [22] concluded that the attenuation of focal osteolytic lesions in VNCa bone marrow images could predict disease status. They analyzed focal osteolytic lesions in VNCa images from patients with active and inactive disease, applying the IMWG criteria, and found a strong negative association between their attenuation in CT and T1 weighted image (T1WI) signal intensity (SI) and a positive correlation with apparent diffusion coefficient (ADC) values from whole-body diffusion-weighted imaging (WB-DWI). The mean attenuation of the lesions in active disease was superior to that in inactive disease (12.4 HU vs. −25.3 HU), and the optimal cutoff point to differentiate them was −21.4 HU (sensitivity, 92%; specificity, 58%). A visual assessment of focal lesions in color-coded VNCa images predicted disease activity with a sensitivity of 57% and specificity of 70%.

Previously, Kosmala et al. [20] proposed a cutoff point of −44.9 HU (sensitivity, 93.3% and 92.4%) in VNCa images to distinguish between normal and infiltrated bone marrow (using MRI reference to select a ROI); however, differentiation between osteolytic and non-osteolytic lesions was not taken into account, nor between active lesions and potentially inactive lesions after treatment.

Concerning the relation between the activity and attenuation of lytic lesions, Fervers et al. published two studies. One evaluated the change in lytic lesions attenuation following RT treatment, with suggestive results of a correlation between active disease and increased cell density [19]. The mean density of focal lesions decreased after RT from 42 HU to 8.5 HU in conventional CT and from −4.5 HU to −53.5 HU, which appears to reflect fatty replacement as a sign of response. Locally progressive lytic bone lesions after RT exhibited greater intralesional attenuation than stable or responding ones, with a median of 32.5 HU vs. 7.0 HU on conventional CT and −3.0 HU vs. −62.5 HU in VNCa images. Cutoff points for the early detection of local treatment failure were established at 20.5 HU in conventional CT and −27 HU in VNCa maps (sensitivity, 83%; and specificity, 74%). The other study [23] compared DECT and monoenergetic CT with FDG-PET/CT as a standard of reference. They proved a positive correlation between metabolic activity and intralesional attenuation in VNCa images, especially in those with high calcium suppression (CaSupp index 25). The cutoff point to differentiate active and inactive lesions was established at −46.9 HU with good sensitivity and specificity (91% and 88%, respectively). Measurements in monoenergetic CT were not reliable for predicting tumoral activity.

Some studies tried to assess non-lytic infiltration by measuring the density at various skeleton locations on VNCa images, avoiding lytic lesions, and comparing the results with MRI. In 2018, Kosmala et al. [24] reported a mean attenuation in VNCa in patients with normal, focal, and diffuse imaging patterns of −65.8 HU, 3.3 HU, and −13.3 HU, respectively, with statistical differences between diffuse vs. normal, diffuse vs. focal, and normal vs. focal patterns. They established cutoff points of −35.7 HU to distinguish between diffuse infiltration and normal marrow (sensitivity, 100%; and specificity, 97%), and −31.9 HU (sensitivity, 97%; and specificity, 99%) for focal infiltration versus normal marrow. In 2021, Brandelik et al. [18] proved a strong correlation between VNCa images with CaSupp index 65 and ADC map, allowing for the differentiation between diffuse infiltration and non-diffuse infiltration. The best correlation with MRI was found in the L3 vertebra with an optimal cutoff point of 3.1HU (sensitivity, 78.6%; and specificity, 88.9%). The optimal cutoff point for the average from L1 to L5 was −1.6 HU (sensitivity, 78.6%; and specificity, 75.0%). They also reported higher sensitivity and specificity (91.3% and 90.9%, respectively) for VNCa color-coded maps than for gray-coded maps (87.8% and 73.5%) for the detection of diffuse infiltration versus non-diffuse infiltration.

Furthermore, Gu et al. [21] compared DECT and laboratory data. Through semi-automatic bone segmentation, they were able to obtain attenuation values of the entire skeleton. They found a strong positive correlation between marrow density values in the VNCa images and biopsy-derived bone marrow plasma cell infiltration percentage, as well as a negative correlation with the hemoglobin level.

### 3.5. Limitations

For the evaluation of bone marrow infiltration, many different measurement sites in the skeleton and diverse qualitative classification criteria have been taken into consideration [18]. Furthermore, measures taken at different periods after therapy have been analyzed, and not all studies have a histological correlation [19,20].

The results could not be replicated because different acquisition technologies (dual-energy and dual-layer) and postprocessing software were employed. As a result, the reconstructed maps and values could differ depending on the CT vendor [24]. Among other factors, the percentage of calcium suppression used varies depending on the software. Moreover, windowing in VNCa maps is not standardized.

The distinction between marrow infiltration and reconversion to hematopoietic bone marrow cannot be made with DECT [25]. Since the amount of hematopoietic bone marrow varies with age and other factors, the optimal cutoff values to detect diffuse infiltration could be variable [24]. Considering the same values for all patients could lead to false positives for the diagnosis of MM.

### 3.6. Summary

WBLDCT is a fast, easy-to-perform, relatively cheap, and useful technique for the diagnosis and follow-up of MM. The inability to distinguish between the presence of hematopoietic bone marrow and tumor infiltration is a limitation of both conventional CT and DECT. However, DECT has shown potential usefulness with good preliminary results in the detection of bone marrow infiltration without osteolysis and the determination of tumor activity and response to treatment in lytic lesions. Higher density in VNCa images has been associated with less fat and higher cellularity, increasing the likelihood of tumor infiltration and active disease. VNCa images can be especially useful for the quantitative assessment of focal lesions with more remnant trabecular bone or imperceptible remineralization that influences the intralesional density measured on conventional CT. Further studies with DECT are needed to reach solid conclusions and provide definitive recommendations.

## 4. WBMRI

IMWG recommends WBMRI for all patients suspected of having myeloma and who underwent LDWBCT or FDG-PET/CT with negative findings [9] due to its recognized high sensitivity [3,10].

WBMRI is a generally well-tolerated technique that offers the additional benefits of assessing skeletal complications, such as spinal canal and/or nerve root compression, and is the most accurate method for differentiating benign from malignant vertebral compression fractures [26]. When WBMRI is not available, MRI of the spine and pelvis is an acceptable alternative to whole-body MRI to provide sufficient bone marrow imaging [9].

Among all imaging techniques, MRI remains the most sensitive and specific for the detection of bone marrow infiltration in a focal or diffuse way before the appearance of osteolytic lesions [27]. The prognostic relevance of focal lesions (size and number) and/or diffuse patterns detected in WBMRI have been demonstrated [26].

### 4.1. MRI Acquisition Protocol

The Myeloma Response Assessment and Diagnosis System (MY-RADS) imaging recommendations are designed to promote standardization and diminish variations in the acquisition, interpretation, and reporting of WBMRI in myeloma [26].

The core clinical protocol for the spine includes T1WI, Short Tau Inversion Recovery (STIR) or T2 Fat Suppressed T2 Weighted Image (T2FSWI). The whole-body protocol includesT1WI Dixon, STIR or T2FSWI and diffusion-weighted imaging (DWI) (b = 50 and 900 s/mm^2^, including ADC maps). Dixon sequences can be used to quantify a signal drop between in-phase and out-of-phase images or map the fat fraction. A signal drop of less than 20% is usually indicative of a significant decrease in the fatty content and may be used as a criterion for the diagnosis of bone marrow-replacing lesions [28].

DWI is an MRI technique measuring the movement of water molecules in the tissue [9]. The different behavior of the molecules compared to the basal scan can be quantitatively assessed using the ADC value, calculated by dedicated software, and also displayed as an imaging parametric map [29]. WBMRI, as currently used, includes DWI sequences that are sensitive to cellular density and viability and are important for disease detection, monitoring, and therapy response assessments. The inclusion of DWI allows for the highly sensitive and quantitative evaluation of soft tissue and bone marrow, which is widely available and quick to perform and interpret. The correlation of ADC values with cell density permits quantitative response assessments before changes in lesion size, as well as evaluations of response heterogeneity [26]. Myeloma cell infiltration of the bone marrow is characterized by higher ADC values (increased water diffusivity) compared to normal bone marrow because of the absence of fat cells, the higher molecular density, and the high cellularity. The evaluation of DWI is based on comparing high *b*-value SI to adjacent muscle SI, but assessments of ADC maps are numeric. ADC values of normal bone marrow are generally below 600–700 μm^2^/s, and viable tumors lie between 700 and 1400 μm^2^/s. Bone marrow ADC measurements have been achieved with a coefficient of variation of 2.8% in myeloma patients, suggesting excellent reproducibility [30].

### 4.2. Disease Patterns

**Figure 7:**
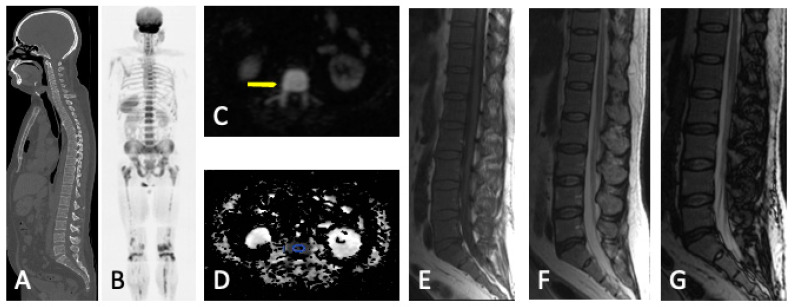
Diffuse MM bone marrow involvement of the axial and peripheral skeleton. No visible lytic lesions in WBLDCT (**A**). Diffuse bone marrow infiltration with high SI in b-900 MR-DWI ((**B**) and arrow in (**C**)) and pathological ADC values (878 μm^2^/s) in the vertebral body (circle in (**D**)). Malignant imaging features with diffuse bone marrow hypointensity in T1WI (**E**) and an SI drop of <20% between the in-phase (**F**) and opposed-phase MRI (**G**).

There are five MRI patterns of bone marrow infiltration in MM:

- Apparently normal bone marrow.
- Focal pattern: Focal myeloma infiltration was defined by circumscribed areas of high SI on STIR and T2WI. These corresponded to areas of low SI or, in a few cases, isointense signal upon an unenhanced T1WI [31]. The definition of focal lesion has evolved lately through the use of sequences such as DWI and Dixon. Therefore, focal lesions are defined as lesions greater than 5 mm hyperintense to background muscle at a *b*-value of 900 s/mm^2^, using ADC maps and confirming these findings with the corresponding Dixon sequences [32].
- Micronodular pattern: The micronodular or variegated or “salt and pepper” pattern presents a widespread heterogeneity with tiny nodular areas of altered diffusion signal (<5 mm) and T1WI hypointensities with preserved normal marrow between them [26].
- Diffuse pattern: Diffuse disease can be suspected from a diffuse decreased signal on T1WI (either iso- or hypointense to intervertebral discs and muscle) and a diffuse increased signal throughout the marrow on T2FSWI, STIR, or high *b*-value DWI. Marrow ADC values above 600–700 μm^2^/s in a nontreated and newly diagnosed patient with MM could be used to increase confidence for the diagnosis of diffuse marrow involvement [33] (Figure 7). Due to potential false-positive findings, diffuse disease in imaging must be supported by bone marrow trephine biopsy [26].
- Mixed pattern: This pattern combines diffuse and focal patterns.

A low tumor burden is usually associated with the normal or micronodular MRI pattern, but a high tumor burden is usually suspected when there is focal or diffuse involvement. In general, ADC values are highest in focal lesions, followed by diffuse bone marrow involvement patterns, red marrow, and yellow marrow [15]. Apparent diffusion coefficient thresholds for diagnosis of diffuse infiltration have been proposed. ADCs greater than 548 μm^2^/s showed 100% sensitivity and 98% specificity for the diagnosis of a diffuse (vs. normal) MRI pattern [34].

### 4.3. Follow-Up Imaging Features

Focal lesions that respond to treatment show a decrease in size and number and a complete or partial decrease in high SI in T2FSWI, STIR, or high *b*-value SI. Other findings include intra- or peritumoral fat within/around focal lesions (“fat dot” or “halo signs”) with an increase in SI in T1WI. Responding diffuse infiltration shows a decrease in high *b*-value SI, an increase in SI in T1WI, and a decrease in STIR.

Evolution from a normal marrow appearance to focal or diffuse patterns, an increase in the number and size of focal lesions, and an increase in both the *b*-value SI and ADC [35] indicate progressive disease. Other findings that show progression are the occurrence of malignant vertebral compression fractures, appearance/extension of extraosseous spread, or soft tissue plasmacytomas.

**Figure 8:**
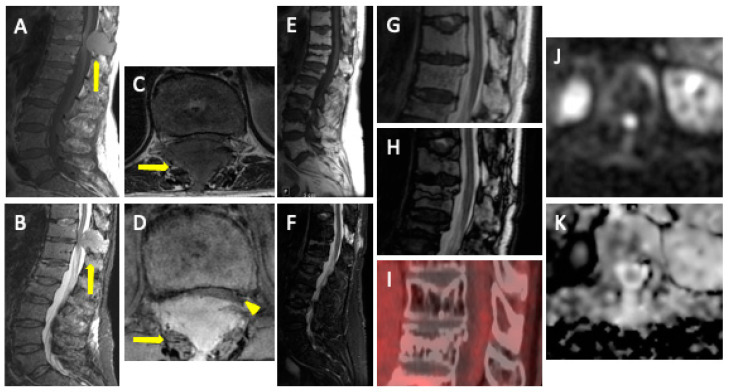
MM with associated plasmacytoma. T1WI (**A**,**C**) and STIR (**B**,**D**) show diffuse involvement of the bone marrow with soft tissue expansive mass (plasmacytoma) in the posterior elements of T11 (arrows) with severe stenosis of the vertebral canal (arrowhead in (**D**)). T1 (**E**) and STIR (**F**) show successful post-treatment changes with fat replacement (hyperintensity in T1) and >20% signal drop between in-phase (**G**) and opposed phase (**H**). There is an absence of FDG uptake (**I**) and a high SI on *b*-900 DWI (**J**), with an increase in ADC (**K**) (“T2-shine-through” effect), which demonstrates a lack of relapse.

Non-responding lesions or relapses show an increase in size and number. ADC measurements offer the capability to quantify disease throughout the skeleton. In the early stage after anti-myeloma therapy (4–6 weeks), the marrow undergoing edema and hemorrhage because of cellular death and vascular congestion results in an increase in ADC and high SI on high *b*-value images (“T2-shine-through” effect) [26] (Figure 8). Lesions must be carefully assessed during this period to avoid mistaking them for progression. With fatty reconversion in the late stage after therapy, a normalization of the bone marrow appearance on the MRI and a decrease in ADC are revealed. The ADC of the marrow of patients in remission and with MGUS is not significantly different from normal age-matched volunteers [36]. The direction of ADC change and cutoff values may be influenced by the timing of measurement, and the optimal timing of DWI after treatment has not been established so far [37,38].

Giles et al. [30], in 2014, studied ADC values to quantify response to treatment. The mean ADC increased in 95% of responding patients and decreased in all non-responders (*p* < 0002). A 33% increase in ADC helped identify response with 90% sensitivity and 100% specificity. Two recent meta-analyses confirmed that a 35% increase in ADC from baseline values is found to classify response post-induction chemotherapy [39], and ADC had a pooled sensitivity of 78% and specificity of 73% in distinguishing responders from non-responders [40]. Zhang et al. [41] studied the correlation between baseline ADC values and survival in MM patients. The mean ADC value of the representative background bone marrow (L3-S1 and iliac bone) was an independent risk factor for both progression-free survival (PFS) and overall survival (OS) when higher than 450 μm^2^/s. 

Limitations of the ADC cutoff values exist due to the ADC dependence on the chosen *b*-values and a lack of standardization of WB-DWI acquisition parameters. The lack of repeatability slows down the development of quantitative imaging biomarkers [42]. DWI is also exquisitely sensitive to trephine tracts, and if trephine tracts have already been performed, WBMRI can provide imaging findings about the location of prior tracts. Alternatively, the WBMRI can be used to select the side for trephine sampling [26,36].

The use of the Dixon also allows researchers to evaluate the degree of adipose re-population of the responding lesions after treatment, therefore adding the ability to discriminate responding lesions compared to lesions resistant to therapy and reducing the false-positive cases in DWI [38]. Koutoulidis [43] studied histogram metrics from ADC and relative fat fraction maps from MRI examinations of 23 patients performed before treatment and after the first treatment cycle. Fat fraction histogram metrics had a higher performance than ADC histogram metrics upon MRI examination for predicting very early therapy response assessment.

Dynamic contrast-enhanced imaging is not usually performed in MM but provides information on bone marrow perfusion, which is systematically altered in different disease stages and can be evaluated in a qualitative and a (semi-)quantitative manner. MM bone marrow infiltration is typically characterized by type 4 curves, and less frequently type 3 or 5 curves (Supplementary Figure S1). Type 1 and 2 curves typically occur in healthy persons or patients with MGUS [35]. Terpos et al. [44] demonstrated a higher wash-in slope for MM as compared to MGUS/SMM. Moreover, they found an inverse correlation between time-to-peak and the Revised International Staging System.

### 4.4. Summary

WBMRI now serves as an imaging tool that has been tailored to detect both focal and diffuse disease in order to quantify burden and response, whilst also assessing mechanical complications. This blend of anatomical and functional imaging is well suited to serve the imaging needs of patients with myeloma and is positioned as a new imaging standard. Conventional MRI has a high sensitivity for bone marrow assessment but cannot safely differentiate between active and inactive lesions. Therefore, FDG-PET/CT has been more widely used, at least for the monitoring of treatment response. Comparative, but mostly retrospective studies, have shown that functional MRI techniques such as DWI, can evaluate tissue cellularity with high sensitivity, which challenges the dominance of FDG-PET/CT in treatment response assessment. Health economic evidence also supports the adoption of WBMRI because, although the upfront costs are higher than those of low-dose CT, the additional benefits result in an equivalent net monetary benefit compared with a negative net monetary benefit for FDG-PET/CT [45].

## 5. PET

### 5.1. PET/CT

#### 5.1.1. Technique and Image Analysis

FDG-PET/CT is a widely used whole-body imaging modality for the detection and staging of tumors, monitoring treatment response, and predicting prognosis in various malignant neoplasms. PET/CT utilizes a radiolabeled glucose analog in which the C-2 hydroxyl group is replaced by the positron-emitting [18F]. [18F]FDG is not further metabolized in the glycolytic pathway, and the molecule becomes trapped intracellularly, allowing for an accurate assessment of glucose metabolic activity [46,47]. The CT component of the examination provides the anatomical localization of metabolic foci and the detection of lytic lesions, whether or not they are associated with increased tracer uptake.

The standardized method for assessing metabolic behavior is visual or qualitative analysis, along with semi-quantitative analysis. The latter uses a voxel-based FDG uptake value known as the Standardized Uptake Value (SUV) proposed by Haberkorn [48]. Haberkorn defines SUV as the ratio of FDG concentration in tissue in Bq/mL to the injected dose in Bq divided by the patient’s body weight in kg: “SUV = FDG (Bq/mL) × 1000/FDG (Bq injected) × weight (kg)” [49]. There are various types of SUVs, with the most commonly used ones in daily practice being SUVmax (maximum value in the ROI) and SUVmean (mean of the values in the ROI).

**Figure 9:**
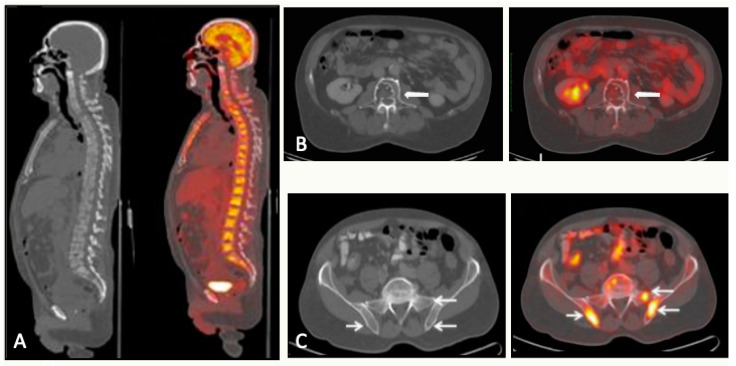
CT and FDG-PET/CT correlation: three different scenarios. (**A**) A 68-year-old man with stage IIIB multiple myeloma with numerous 18FDG-avid lytic lesions in the axial skeleton. (**B**) A 71-year-old man with stage IIIB multiple myeloma. Lytic lesion in L3 without FDG uptake (arrow). (**C**) A 72-year-old man with stage IIB multiple myeloma. Multiple hypermetabolic foci in the sacrum and both ilia without correspondence in CT scan (arrows).

Several factors influence and result in variations in the SUV measurement, including blood glucose levels, the time elapsed from injection to the start of image acquisition, equipment calibration, the patient’s weight, the injection technique, the ROI of interest, the reconstruction method employed, and the size of the matrix. To date, there are no established SUV normality values, nor is there a cutoff point at which it is considered pathological. This is because radiotracer uptake is multifactorial and depends on factors such as the location of the ROI, the radiotracer’s affinity for tissue in each pathology, and the size of lesions detected by the equipment, leading to a reduced uptake in lesions smaller than 10–15 mm due to spatial resolution, among other factors (Figure 9).

#### 5.1.2. Response Measurement

FDG-PET/CT is currently the imaging modality of choice for the assessment of therapeutic response [9]. Its capacity to distinguish between active and inactive residual disease and ability to offer a precise anatomic characterization of bone and extra-medullary lesions are key factors in the widespread adoption of this hybrid modality in routine practice.

Following first-line therapy, FDG-PET/CT has shown a strong prognostic value in several prospective trials of newly diagnosed patients. In 2018, Davies et al. [50] demonstrated that, at day 7 of therapy, patients with complete focal lesion signal suppression revert to the same prognosis as those with no lesions at diagnosis. At later time points, the continued suppression of signal remains prognostically important, emphasizing that the therapeutic goal should be the complete suppression of focal lesions. Similar results were obtained by Zamagni et al. [51] in a cohort of 192 newly diagnosed MM patients after induction therapy and double autotransplantation. SUV values > 4.2, indicating the persistence of FDG-avid bone lesions following induction therapy, were an early indicator of a shorter PFS. In another prospective trial, SUV normalization (defined as uptake ≤ liver background) before maintenance was associated with improved PFS and OS [52]. 

#### 5.1.3. Minimal Residual Disease

In 2016, the IMWG updated its criteria regarding minimal residual disease, and thus FDG-PET/CT is required if minimal residual disease (MRD)-negative status is reported [7]. WB-DWI and FDG-PET/CT may overcome some of the limitations of molecular techniques and have been used to redefine complete response in patients with MRD-negative bone marrow.

The multicenter phase II randomized FORTE trial in newly diagnosed MM demonstrated the complementarity of FDG-PET/CT with bone marrow MRD techniques [53]. Concordant negativity between FDG-PET/CT and MRD bone marrow techniques should be evaluated as a surrogate for outcome prediction [54]. 

Data from the IFM2009 trial demonstrated equivalent efficacy for MRI and FDG-PET/CT in the detection of bone lesions at diagnosis [52]. MRI normalization did not translate into any improvement in PFS or OS in this study in contrast to FDG-PET/CT. The usefulness of MRI for the assessment of residual disease after therapy remains unclear at this time due to the lack of sufficient data. On the other hand, the potential role of DWI in MRD detection was investigated by Rasche et al. [55], who performed flow cytometry, FDG-PET/CT, and DWI in 168 patients, achieving complete response after first-line or salvage treatment. In this study, DWI detected more patients with residual focal lesions than FDG-PET/CT (21% vs. 6%, respectively).

#### 5.1.4. Alternative PET Tracers

In patients with MM, several tracers other than [18F]FDG targeting other biological processes have been successfully evaluated. Studies have been conducted with lipid tracers such as [11C] or [18F]-choline, and [11C]-acetate; and amino acid tracers like [11C]-methionine [56,57]. The CXCR4-targeting PET tracer (68 Ga-CXCR4) in MM patients revealed that CXCR4 expression is usually found in advanced MM and is considered an adverse prognostic indicator [58].

#### 5.1.5. FDG-PET/CT vs. WBMRI

**Figure 10:**
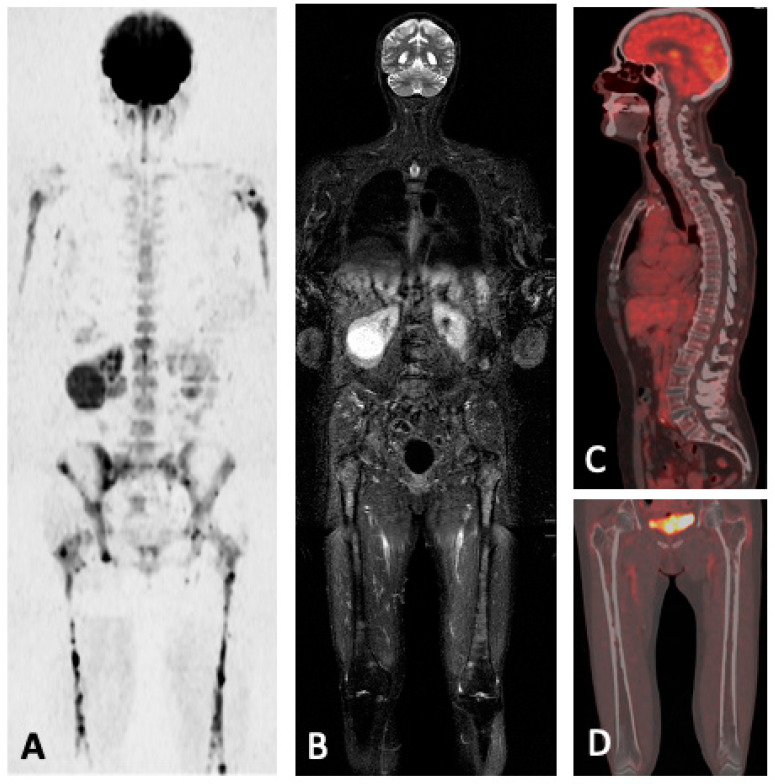
A 70-year-old woman with multifocal involvement of MM in the axial and peripheral skeleton. Multiple lesions with high SI in *b* = 900 DWI (**A**) and high SI in STIR (**B**). Follow-up FDG-PET/CT (**C**,**D**) shows successful post-treatment changes with no pathological FDG uptake.

Comparative studies have suggested that WBMRI is more sensitive than FDG-PET/CT for detecting bone marrow infiltration in MM diagnosis due to its high spatial resolution and sensitivity to diffuse infiltration [59]. FDG-PET/CT correlates well with the patient’s clinical response, and 18F-FDG PET suppression precedes normalization in MRI (Figure 10). Nevertheless, MRI is also a valid tool to assess treatment response, because the resolution of lesions in MRI after therapy is associated with a favorable prognosis, although it takes longer for responding lesions to disappear than in PET [31].

In the meta-analysis (12 studies) from Rama et al., the pooled sensitivity and specificity were 90% and 56% for whole-body MRI versus 66% and 81% for FDG-PET/CT [60]. Yokoyama et al. [61] performed a meta-analysis (six studies) comparing both techniques and concluded that FDG-PET/CT had higher sensitivity and greater ability to detect the treatment assessment of MM. Several studies included in the meta-analysis were limited by the lack of use of DWI sequences, which are known to improve the ability to distinguish viable from non-viable residual marrow abnormalities after treatment [62]. 

Recently, a prospective comparison of WBMRI, including DWI, with FDG-PET/CT by Messiou et al. [32] in 60 patients with newly diagnosed MM or relapsed MM showed a higher sensitivity of WBMRI in detecting both focal and diffuse disease. Studies have found higher lesion conspicuity on DWI when compared to FDG-PET/CT in patients with MM (sensitivity of DWI has been reported to be 77%, compared with 47% for FDG PET/ CT) [31]. DWI with suppression of background body signals can detect lesions more reliably with high sensitivity than FDG-PET/CT, albeit at the expense of specificity. DWI had a pooled sensitivity of 78% and specificity of 73% in distinguishing responders from non-responders, emphasizing the importance of DWI for treatment response assessment in patients with MM [40].

Paternain et al. [63] studied the correlation between the ADC and SUVmax of MM lesions. They concluded that there was indeed a strong, negative, and significant correlation (r = −0.603). After treatment, the mean ADC in lesions from responders was significantly higher than in non-responders (1585.51 × 10^−6^ mm^2^ vs. 698.17 × 10^−6^ mm^2^/s). SUVmax of the same lesions was significantly lower in responders than in non-responders (2.05 vs. 5.33). Despite significant advances in the utility of ADC for MM follow-up imaging, current guidelines consider FDG-PET/CT as the preferred technique for monitoring treatment response in MM [62,64]. 

### 5.2. FDG-PET/MRI

Whole-body FDG-PET/MRI is an emerging hybrid imaging technique that offers the opportunity to combine in one examination the most sensitive information on morphology, bone marrow cellularity and vascularization, and metabolic activity; however, the real upgrade in patient care, given the cost and the need for double expertise, is still under investigation [65]. Considering that FDG-PET/MRI is an emerging technique, neither the National Comprehensive Cancer Network nor the IMWG, the two primary organizations that publish guidelines in this area, support its use in staging or after treatment. The two underlying modalities, FDG-PET and MRI, have each been individually suggested for use in a variety of settings [66].

There has been little academic work specifically on the use of FDG-PET/MRI for MM. Sachpekidis et al. [67] showed that FDG-PET/MRI exhibited an equivalent performance to FDG-PET/CT regarding the detection of myeloma skeletal lesions. SUVmax values of MM lesions were lower on FDG-PET/MRI but tightly correlated with FDG-PET/CT (allowing the possibility that FDG-PET/MRI can still be used with a conversion factor). The complementarity between WBMRI and FDG-PET can be a key advantage of FDG-PET/MRI technology, especially in assessing treatment response and MRD. Rasche et al. [55] studied MRD in newly diagnosed patients achieving complete response, and they showed that the most sensitive approach is using both DWI and PET, allowing for the detection of focal residual disease in 24% of these patients.

The choice between FDG-PET/MRI and FDG-PET/CT may depend on the institution and individual patient circumstances. FDG-PET/MRI is chosen in cases where there are no contraindications for MRI, aiming to leverage the advantages of both modalities for more precise staging and treatment response assessment. At present, billing and operational workflow are the bigger challenges of hybrid FDG-PET/MRI imaging.

### 5.3. Summary

FDG-PET/CT is considered the preferred imaging technique for evaluating and monitoring metabolic response to therapy because of its ability to distinguish between active and inactive disease. MRI has the highest sensitivity in detecting lesions, while FDG-PET/CT has a major impact on prognosis. Note, however, that false-negative and false-positive results may occur with the use of FDG-PET/CT, as it has limitations in assessing diffuse BM infiltration and differentiating MM lesions from inflammatory or infectious lesions. The standardization of FDG-PET/CT is ongoing through an accurate descriptive analysis based on Deauville criteria applied to lymphomas.

FDG-PET/MRI technology represents a valuable emerging imaging modality in the management of MM, offering the capability to assess disease burden throughout the body, monitor treatment response, and detect early relapses. Its selection may depend on the clinical situation and medical center preferences, although it is not widely implemented, and, currently, it is not included in clinical guidelines.

## 6. Plasmacytoma and Extramedullary MM

Plasmacytoma is a tumor caused by the clonal proliferation of plasma cells. It can manifest as a primary neoplasm (solitary plasmacytoma) or secondary to dissemination of MM. These two conditions are histologically identical and are a subset of plasma cell dyscrasias. The main distinction is that, in primary plasmacytoma, the bone marrow biopsy is normal, containing less than 10% plasma cells, and there are no systemic symptoms associated with myeloma (renal insufficiency, hypercalcemia, anemia, or bone lesions) other than the plasmacytoma itself [68]. 

**Figure 11:**
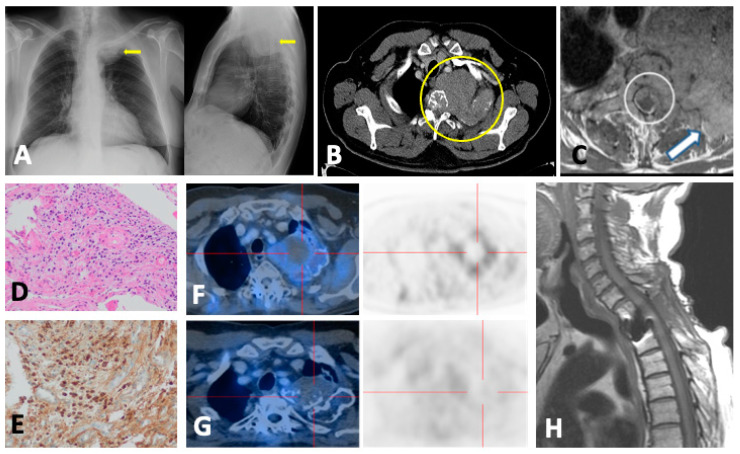
A 68-year-old male who was admitted to the emergency department for subacute left infraclavicular pain that radiates to the left upper limb. (**A**) Chest X-ray revealed a mass in the left pulmonary apex (arrows). (**B**) Chest CT confirmed the presence of a mass in the left pulmonary apex (circle) with mediastinal extension, second left rib destruction, infiltration, and partial destruction of T2 vertebra with probable epidural soft tissue mass and soft tissue component in the adjacent extra thoracic musculature. The differential diagnosis was Pancoast tumor, metastasis of unknown neoplasm, lymphoma, and multiple myeloma/plasmacytoma. (**C**) Gadolinium-enhanced T1FSWI confirmed the solid nature of the lesion (arrow) and the presence of an epidural soft tissue mass causing cord compression (circle). (**D**) Histological (hematoxylin–eosin) and (**E**) immunochemical examinations revealed a clonal proliferation of plasma cells consistent with plasmacytoma. (**F**) Staging FDG-PET/CT evidenced hypermetabolism of the apical lesion with probable necrotic/hemorrhagic central component without distant skeletal uptakes or additional soft tissue masses. The patient was treated with decompressive surgery, RT, and chemotherapy (T1WI in (**H**)). (**G**) One-year follow-up PET/CT showed metabolic response with persistence of tumor.

Three different types of solitary plasmacytomas can be identified based on the location and number of lesions: solitary extramedullary plasmacytoma, solitary bone plasmacytoma, and multiple solitary plasmacytomas, which occur when there are multiple plasmacytoma lesions but no MM (IMWG, 2003). Solitary plasmacytomas are relatively rare, accounting for only 3% of all plasma cell neoplasms [69] (Figure 11). A majority of solitary plasmacytomas (70%) are osseous (SBP), with vertebrae, ribs, the skull, and pelvic bones being the most often affected bones [69,70]. Extramedullary plasmacytomas preferentially localize in the nasopharynx and oropharynx, although cases of plasmacytomas have also been described in the gastrointestinal tract, skin, lymph nodes, breast, gallbladder, thyroid gland, parotid gland, and central nervous system [71].

Due to the rarity of the illness, case reports and case series make up the majority of the literature on the imaging diagnosis of plasmacytomas. There are currently very few studies that compare MRI with FDG-PET/CT for the initial assessment of this condition.

In a prospective study of patients with plasmacytoma, FDG-PET/CT was found to be superior to MRI in both diagnosis and follow-up. It should be noted that contrast-enhanced MRI was not used; instead, MRI of the spine and pelvis was employed. FDG-PET/CT detected plasmacytomas in soft tissue, skull, ribs, and extremities that were outside the anatomical field of view of MRI [72]. In another retrospective study of patients with solitary plasmacytoma, FDG-PET/CT was once more superior to MRI of the spine and pelvis for detection and follow-up [73]. The only study to date that compares contrast-enhanced MRI and FDG-PET/CT for the diagnosis of plasmacytomas [74] concludes that there are no significant differences, although its statistical power is limited by the sample size (n = 34). The IMWG recommends FDG-PET/CT as the first imaging test for patients with suspected extramedullary plasmacytoma to rule out other lesions. The British Society of Hematology, in contrast, defers to clinical judgment when determining which test to use [75].

The analysis of extramedullary plasmacytomas is more challenging because imaging tests alone cannot differentiate these lesions from more common malignant tumors, such as metastases or carcinomas [68]. As a result, histological confirmation is required, and WBMRI and FDG-PET/CT should be used to focus on early detection, which may influence treatment and prognosis [70]. Depending on the location, other imaging tests may be useful (in this study, plasmacytomas in breast, testis, and lymph nodes were also evaluated with ultrasound). Furthermore, it is important to note that FDG-PET/CT cannot reliably distinguish between MM and multiple solitary plasmacytomas, so histological confirmation is essential [76].

The most frequent location of the SBP is the thoracic vertebral bodies. The typical presentation is a lytic or mixed lesion with relative respect of the cortical bone in a curved manner and thick intralesional struts conforming to the distinctive “mini-brain” appearance. Variable degrees of collapse can result from partial destruction of the vertebral endplates. Presentations with a multicystic “soap bubble” appearance mimicking the hemangioma of a pure lytic lesion are less common. Although uncommon, sclerotic plasmacytoma can be found in patients with POEMS syndrome [77,78]. It has low SI on T1WI, high SI on T2WI, and homogeneous marked contrast enhancement. Extraosseous plasmacytoma manifests as a homogeneous soft tissue mass with avid uptake of radiotracer on FDG-PET/CT [70]. Radiation therapy and total surgical excision are the mainstays of treatment.

Furthermore, up to 16% of MM patients also have an extraosseous spread of the disease, which worsens their prognosis. It is more common in younger patients and non-secreting and IgD subtypes [79]. Extraosseous symptoms following autologous or allogenic stem cell transplantation have been reported to occur more frequently [80]. The higher rate of extraosseous recurrence in these patients has been suggested to be caused by extraosseous locations acting as sanctuary sites that are not successfully treated by stem cell transplantation [79].

**Figure 12:**
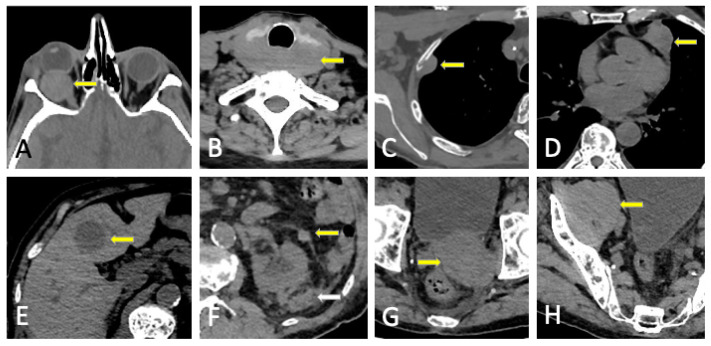
Extraosseous spread of MM with arrows. (**A**) Right orbital mass, (**B**) retrothyroid conglomerate nodes, (**C**) pleural soft-tissue mass, (**D**) pericardial mass, (**E**) hypodense hepatic focal lesion, (**F**) soft-tissue confluent masses in perirenal fat and fascia, (**G**) pelvic soft-tissue mass, and (**H**) enlargement of right psoas secondary to tumoral infiltration.

Extraosseous disease and extramedullary plasmacytomas manifest on CT as homogeneous soft tissue density masses without necrosis or calcification. The masses show the same behavior in WBMRI as bone lytic lesions in T1WI and T2WI and also avid FDG uptake at PET/TC [79]. Diffuse infiltration of the spleen and liver and adenopathy or conglomerates that simulate lymphoma are the most common findings. Renal or perirenal masses, intestinal wall thickening, or mesenteric nodules that mimic lymphoma and diffuse peritoneal involvement simulating carcinomatosis can also be seen quite frequently (Figure 12). The most common thoracic finding is pulmonary nodules. However, it must be taken into account that the involvement of any organ or tissue is possible [79,81].

## 7. Structured Reporting

The following information should ideally be available to radiologists at the time of reporting: time of initial diagnosis or suspected diagnosis, serum paraprotein levels and light chain levels and trephine status (if performed, including site), symptomatic sites including any clinical indication of cord or nerve root compression, first line or relapse state, current treatment, history of autograft or allograft transplant, details of previous radiation therapy or surgical interventions including vertebroplasty, granulocyte colony-stimulating factor or steroid administration, and minimal residual disease status [26]. In general terms, the report should include the technique performed, use of contrast, field of view, comparison with previous image studies if available, quality of the examination, and limitations. Depending on the technique used, some other specifications (morphometric data of the patient, time of injection, amount of contrast or radiopharmaceutical used, etc.) of greater relevance in FDG-PET/CT will be added (Table 2).

Regarding LDWBCT, in 2018, a report by the Bone Group of the IMWG was published to provide practical recommendations for the acquisition, interpretation, and reporting of WBLDCT in patients with MM and other plasma cell disorders [14].

MY-RADS imaging recommendations recently proposed the criteria for the response assessment category (RAC) with a 5-point scale to standardize response assessment after therapy, but this score still needs to be validated [26]. A recent study supports the applicability of MY-RADS recommendations after autologous stem cell transplantation; RAC criteria were able to independently stratify patients and to better predict their prognosis, and the combined use of WB-DWI with flow cytometry allowed for a more precise evaluation of minimal residual disease (MRD) [38]. Paternain et al. [63] showed a strong, negative, and significant correlation between the ADC and SUVmax of all the target lesions and a strong agreement of the IMWG response criteria with both MRI and FDG-PET/CT. 

To standardize and harmonize the reporting and interpretation of FDG-PET/CT in routine practice and clinical trials, new visual descriptive criteria have been defined (Italian Myeloma Criteria for PET Use, IMPeTUs) [82], although they are still yet to be validated. These criteria are a combination of visual assessment of FDG uptake, using the 5-point Deauville score (initially designed for lymphoma) and typical MM-related features, such as focal bone lesions, diffuse bone marrow uptake, fractures, and extramedullary lesions. The current IMWG criteria [7] strictly define imaging response as the disappearance of activity or residual activity below the mediastinal blood pool (analogous to a Deauville score of 1 or 2). Residual FDG uptake in localized lesions and bone marrow below the liver background (corresponding to a Deauville score of 1 to 3), as described by Zamagni et al. [83], may eventually replace the existing IMWG response criteria.

**Table 2:** Recommendations on the imaging reporting in monoclonal plasma cell disorders.

	LDWBCT	WBMRI	FDG-PET/CT
First diagnosis	Lytic lesions: Size measurements of main lesions; if less than 10, specify number and location, if more, describe the most relevant (craniocaudal order; alternatively, largest lesion first); associated soft tissue masses Paramedullary/extraosseous disease (location, extension, and complications) Intramedullary deposits in long bones (extension and endosteal scalloping). Risk of fracture in weight-bearing bones Vertebral fractures (possible etiology, instability, and risk of cord/root compression) Incidental relevant findings (including previous musculoskeletal surgery)	Disease involvement: measurement of 5 lesions and/or associated pattern of marrow infiltration (normal, focal, focal on diffuse, diffuse, micronodular) Extraosseous disease: size of paramedullary or extramedullary involvement Infiltration of long bones Vertebral fractures: Characterize as benign vs. malignant, instability (SINS) [84], cord (ESCC) [85]/nerve root compression Posterior iliac crest disease Incidental findings (i.e., avascular necrosis)	Localization (bone, paramedullary, and extramedullary) extent and intensity of pathological radiotracer accumulations (SUVmax) in relation to (suspected) MM Relevant findings in CT and correlation with metabolic activity Report accumulation as mild, moderate, or intense and compared to the background uptake (liver parenchyma) IMPeTUs (summarized) [80]: -Diffuse bone marrow uptake (Deauville 5 grades). A if limbs and ribs involvement -Focal bone lesions by number group -Skull, spine, and extraspinal -Target (hottest) focal bone lesion (Deauville 5 grades) -Lytic lesions at associated CT by number group -Fractures -Paramedullary disease -Extramedullary disease (specify site) Target (hottest) extramedullary lesion (Deauville 5 grades)
Follow-up	New lytic lesions/soft tissue masses Change in features of known lytic lesions Change in the intramedullary attenuation of long bones Risk of fracture in weight-bearing bones Evolution of paramedullary/extraosseous disease New fractures and possible etiology Evolution of known fractures (stability, healing, and osteosynthesis complications) Incidental relevant findings (including previous musculoskeletal surgery)	5-point scale MY-RADS response assessment categories [26] 1: Highly likely to be responding -Unequivocal ↓ in number—size FL/soft tissue (RECIST: PR, CR) -≥40% ↑ in ADC and ↓ in high *b*-value SI -ADC: from ≤1400 to >1400 -Intra- or peritumoral fat of previous FL/DI 2: Likely to be responding -Slight ↓ in number—size FL ->25% and <40% ↑ in ADC and ↓ in high *b*-value SI -ADC: from ≤1000 to <1400 3: Stable 4: Likely to be progressing -Equivocal new FL -↑ in high *b*-value SI and ADC < 1400 -Relapsed disease -Spinal canal stenosis without symptoms 5: Highly likely to be progressing -Unequivocal new or ↑ in number—size FL/DI/soft tissue (RECIST: PD) -New FL with ADC: 600–1000 -New fracture/cord compression requiring intervention	Evolution of number or extent and intensity of known pathological radiotracer accumulations Evolution of previous relevant findings in CT New accumulations in relation to the suspected evolution/relapse of MM New relevant findings in CT and correlation with metabolic activity IMPeTUs (same as first diagnosis)
Conclusion	Clear and concise impression Recommendations for follow-up or need for additional test		

SINS, Spinal Instability Neoplastic Scale; ESCC, Epidural Spinal Cord Compression Scale; MY-RADS, The Myeloma Response Assessment and Diagnosis System (still need for validation); RECIST, Response Evaluation Criteria in Solid Tumors; PR, partial response; CR, complete response; PD, progressive disease; FL, focal lesion; SI, signal intensity; DI, diffuse infiltration; ADC units, μm^2^/s; ↑: increase; ↓: decrease.

## 8. Pitfalls and Differential Diagnosis

### 8.1. Diffuse Imaging Findings

Red marrow reconversion is very frequent in MM, occurring in association with anemia, chemotherapy, bone marrow-stimulating factors, or multiple factors. This can obscure the presence of focal bone lesions or lead to false-positive results in MRI and predominantly in CT or FDG-PET/CT, as it mimics diffuse infiltration [9,26]. In CT, the presence of endosteal thinning suggests a tumoral etiology, while scarce conspicuity and poor definition of the edges favor red marrow. However, it is necessary to perform additional tests (MRI, PET-CT, biopsy, etc.) to differentiate these two entities [14]. Red bone marrow hyperplasia in the setting of anemia may cause changes in ADC and fat fraction values that could confound the assessment of disease severity. In particular, red marrow hyperplasia may be associated with an increase in ADC values that mimics either focal lesions or diffuse disease.

Differentiation of red marrow reconversion and true lesions remains a challenge in patients with MM and anemia. Dong et al. [86] studied the utility of the MY-RADS total burden score, ADC, and fat fraction from WBMRI in predicting early treatment response in patients with newly diagnosed MM and comparing the utility of these measures between patients with and without anemia. They concluded that a low total burden score, low ADC, and high fat fraction from WBMRI may predict deep response in patients with MM, although only among those without anemia.

Strategies for mitigating false-positive results include direct correlations with morphologic appearances, including T1WI, Dixon images, and DWI/ADC [26]. Both WBMRI and FDG-PET/CT should be avoided in the days following granulocyte-colony stimulating factor administration; but if this is not possible, diffuse abnormalities should be interpreted with caution [35]. The differential diagnosis of diffuse bone marrow infiltration must include mastocytosis, myelofibrosis, lymphoma, and leukemia. 

### 8.2. Malignant Focal Lesions

The radiological diagnosis between metastasis or lymphoma and MM based on imaging findings, especially when it comes to incidental findings, can be challenging, as both can show osteolysis and soft tissue masses and also the same behavior at MRI. A mixed appearance of bone lesions or a sclerotic rim in lytic lesions excludes the diagnosis of MM if untreated. Early involvement of pedicles and sclerotic lesions favor the possibility of bone metastasis rather than MM. The involvement of the intervertebral disk and adjacent vertebrae favors MM or plasmacytoma over metastasis [87].

### 8.3. Benign Entities

Not all hyperintense bone lesions on high *b*-value images are malignant. Causes for apparent high *b*-value focal bone lesions that are false positive for cancer include bone marrow edema caused by fractures, osteoarthritis, infection, bone infarcts, vertebral hemangiomas, chondromas, cysts, focal fat–poor bone marrow, and artifacts around metal implants. To avoid misinterpretations arising from the visual assessment of SI, it is essential to correlate *b*-value findings with ADC maps, as well as an analysis of the morphological features from the associated conventional WBMRI sequences. 

[18F]FDG is a non-specific marker that can accumulate in coexisting infectious or inflammatory disorders, degenerative joints, or other benign conditions, leading to false-positive results. The absence of hexokinase in at least 10% of MM patients is the primary factor in false-negative FDG-PET/CT results. In this subgroup of baseline false-negative patients, MRI and other PET tracers targeting alternative pathways should be used [88].

**Figure 13:**
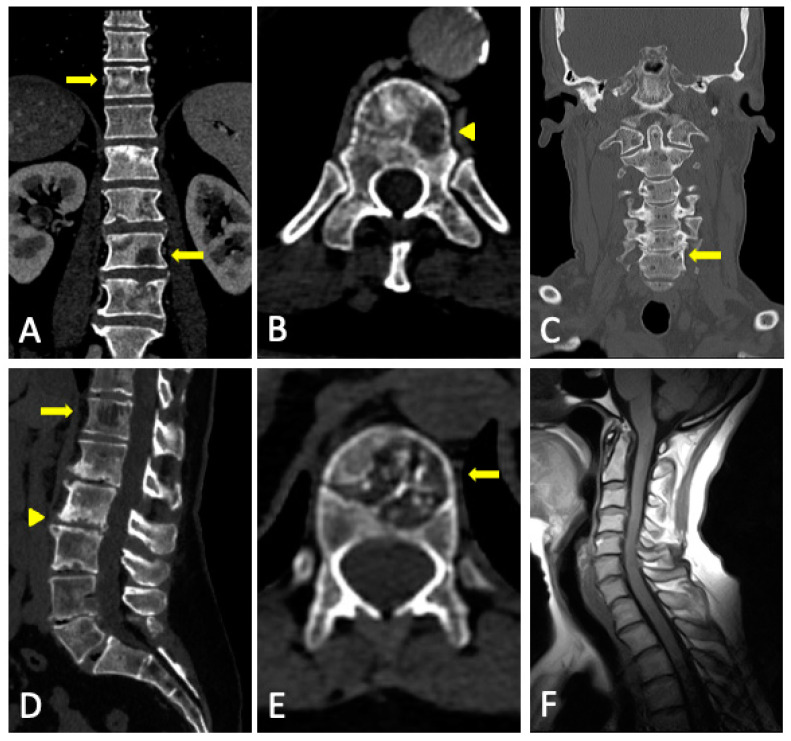
Differential diagnosis in conventional CT. Multiple osseous lesions (arrows in (**A**)), some of them with mixed pattern and sclerotic rim (arrowhead in (**B**)) corresponding to lung cancer metastases, incompatible with untreated MM. Angioma in T12 vertebral body (arrows in (**D**,**E**)) with intralesional fat and intralesional thick trabeculae conforming to the typical “jail bar pattern” in sagittal reconstruction (**D**) and “polka dot pattern” in the axial plane (**E**). Degenerative disc disease in lumbar spine with multiple Schmörl nodules (arrowhead in (**D**)). We present a case of osteoporosis with multiple millimetric lytic lesions in coronal reconstruction of cervical spine (**C**), some of them with sclerotic rim (arrow in (**C**)) with a normal signal of the bone marrow in T1WI sequence (**F**) and no findings of hematologic cell dyscrasia in laboratory tests.

Hemangiomas are the most frequent benign tumors in the spine. Atypical and aggressive angiomas can mimic plasmacytoma since they are lytic lesions with a predominant vascular component that confers a hypointense signal on T1WI and a hyperintense signal on T2WI and STIR with variable enhancement intensity after the administration of paramagnetic contrast. In addition, aggressive hemangiomas can exhibit cortical rupture, insufflation, and perivertebral soft tissue masses. The presence of thick intralesional trabeculae creating the typical “polka dot” pattern in axial images and macro- or microscopic fat are the keys to diagnosis (Figure 13). DWI sequences can help, as they exhibit a T2 shine-through effect in ADC maps contrary to untreated myeloma lesions. The ADC of hemangiomas is significantly higher than that of focal active myeloma deposits: 1.085 × 10^−3^ mm^2^/s compared with 0.682 × 10^−3^ mm^2^/s. Therefore, the ADC map combined with appearances on sagittal T1WI and T2WI imaging of the spine should avoid misdiagnosis as active myeloma deposits [28]. Upon the performance of FDG-PET/CT, they may show mild uptake, unlike MM [89].

Patchy osteoporotic involvement can mimic MM on conventional CT with pseudolytic lesions or cortical thinning, although osteoporosis remains occult in MRI studies and does not show increased metabolism on FDG-PET/CT [90].

Schmörl’s hernias are extremely common. They can be distinguished from MM lesions by their subchondral location, sclerotic ridge on CT, and associated signs of degenerative disc disease.

The presence of intralesional fat leads to alternative diagnoses in untreated patients with suspected myeloma; however, in treated MM patients, it can reflect a total or partial response [14].

## 9. Complications

In the first year following diagnosis, 40% of people with MM develop fractures, and up to 60% of them do so at some time during the illness [15]. As lytic lesions in MM have a particular behavior that is different from that of metastatic lesions, a novel specific scoring system was recently proposed for estimating the fracture risk in patients with MM (Supplementary Figure S2) with better sensitivity and similar specificity than the classic Mirels scoring system, more adequate for metastases [91]. This new scoring method considers lesion latency and location within the bone, in contrast to the Mirels system, and may serve as a more effective screening tool to determine which patients would benefit from further radiologic or orthopedic evaluation based on a skeletal survey.

CT multiplanar reconstructions enable better visualization of vertebral fractures, as well as osteolysis in weight-bearing bones and assessment of fracture risk and spine instability [14]. MRI is the gold standard for the differentiation between benign and malignant osteoporosis or fractures and the diagnosis of cord compression. 

Benign vertebral fractures can be caused by progressive osteoporosis, which is regularly observed in MM due to the combination of senile and cortisone application-induced osteoporosis. Benign vertebral compression features in MRI are normal posterior element signal, retropulsed bone fragments, preserved normal marrow signal, fracture line, fluid sign, loss of SI on opposed-phase and no restricted diffusion (high ADC), a band-like area of bone edema, and coexisting healed benign vertebral fractures at other levels [92].

CT findings that favor malignant causes are bone destruction, soft tissue masses, and involvement of posterior elements in the spine. In contrast, vacuum phenomena, sharp fractures, and retropulsion of the posterior wall favor insufficiency fractures. Malignant MRI features include multifocal lesions, abnormal posterior element signal, convex posterior border, soft tissue mass, diffuse replacement of normal marrow signal, ratio of opposed-phase to in-phase SI > 0.8, and increased restricted diffusion (high *b*-value SI and intermediate ADC) [92].

**Figure 14:**
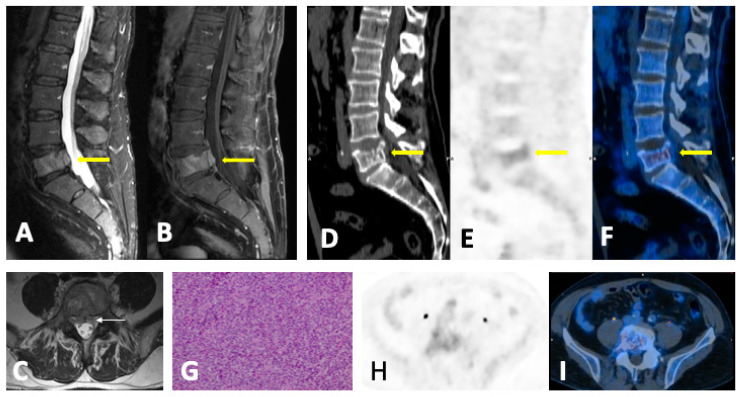
A 50-year-old patient with L5 vertebral body fracture secondary to diffuse tumor infiltration with the collapse of the upper plateau, posterior wall retropulsion, and anterior epidural soft tissue mass (arrows (**A**–**F**)), causing thecal sac indentation. MRI shows an L5 vertebral body malignant lesion with soft tissue expansion in the epidural anterior space in sagittal Dixon “only water” T2w (**A**) and contrast enhancement in sagittal gadolinium-enhanced T1FSWI (**B**) and axial T2w (**C**). The lack of FDH uptake in the L5 level can be observed in sagittal (**D**–**F**) and axial (**H**,**I**) FDG-PET/CT reconstructions, making it difficult to determine a pathologic fracture diagnosis. Histologic sample with hematoxylin–eosin staining diagnostic for plasmacytoma (**G**). The immunohistochemistry revealed monoclonal lambda light chains in plasma cells. The patient was treated with radiotherapy.

In FDG-PET/CT, the SUV is significantly higher in malignant than in benign compression fractures, and cutoff SUVs for malignant fractures ranged from 3 to 4.7 or 2 standard deviations above liver SUV. However, case reports have shown benign fractures with much higher-than-expected SUVs. Therefore the precise role of FDG-PET/CT in the imaging of benign and malignant vertebral compression fractures has yet to be defined [92] (Figure 14). Moreover, the identification of skull lytic lesions is a limitation of FDG-PET/CT.

Vertebral lesions can create spinal column instability and neural compression that can potentially result in significant pain, devastating neurologic consequences, or both. The involvement of more than 50% of the vertebral body and/or posterior elements increases the risk of instability [13]. The Spinal Instability Neoplastic Scale (SINS) is a classification system that includes five imaging criteria and one clinical criterion and can accurately discriminate between stable (0-to-6 points), potentially unstable (7-to-12 points), and unstable (13-to-18 points) (Supplementary Figure S3). Guidelines recommend consultation with a spine surgeon for all patients with a SINS of 7 or greater [93,94]. Up to 30% of MM patients are expected to experience spinal cord compression during their disease [95]. The presence and severity of the cord compression can be classified with the Epidural Spinal Cord Compression Scale (ESCC) on T2WI on MRI. The ESCC classification is used to differentiate no or low-grade ESCC (0-1c) from high-grade ESCC (2-3) (Supplementary Figure S4) [83]. High-grade ESCC and mechanical instability are considered absolute indications for surgery and/or radiotherapy. In these patients, minimal access approaches and targeted tumor excision and ablation techniques reduce the surgical risk and accelerate postoperative recovery. A lower SINS, indicating spinal stability, is associated with a complete pain response to radiotherapy [96].

Extensive lytic lesions in weight-bearing bones require orthopedic consultation to evaluate the need for prophylactic osteosynthesis, mainly intramedullary nailing, as it may require a shorter operative time and hospital stay and better recovery.

## 10. Future Directions

### 10.1. Photon-Counting CT

A novel technology in clinical development is the photon-counting detector (PCD) CT, where X-ray energy is directly converted into electrical impulses. Information about the energy of each photon is recorded based on the magnitude of the electrical signal generated from photon events. PCD-CT allows for the generation of multi-energy images with up to 47% lower image noise or ultrahigh spatial-resolution images with lower radiation doses, compared to the currently used energy integrating detector (EID) CT [97].

A recent study by Winkelmann et al. [98] which included 50 MM patients compared subjective and objective image quality of bone microstructure and disease-related abnormalities on the PCD-CT compared to the EID-CT. The results of this study show significant qualitative and quantitative image quality improvement on the PCD-CT. PCD-CT allowed for easier and more accurate delineation of bony trabeculae, as well as the sharpness of their borders and the transitions between the cancellous bone and focal lytic bone lesions. Interestingly, this trend stayed unimpaired even in bone lesions smaller than 5 mm. This study concluded that the application of PCD-CT is superior to EID-CT in clinical workup of multiple myeloma patients.

Furthermore, Baffour et al. [99] proved that a clinical PCD-CT in ultra-high-resolution mode, both with and without a deep learning noise reduction algorithm, performed better than an EID-CT in displaying multiple myeloma lesions. When compared to EID-CT systems with matched reconstruction parameters (2 mm slice thickness), the images from the PCD-CT clearly showed reduced noise, lytic lesions, intramedullary lesions, and fat attenuation. Additionally, one or more lytic lesions were depicted on thinner PCD-CT images (0.6 mm section thickness) in 21 of 27 participants.

The superior spatial resolution, lower image noise, and better dose efficiency of PCD-CT ushers in a new era of sensitive CT evaluation of myeloma bone disease.

### 10.2. Artificial Intelligence

In terms of LDWBCT for the diagnosis of MM, artificial intelligence is being developed to generate tools that automatically perform segmentation and bone subtraction both in monoenergetic CT and DECT to recognize focal lesions, allowing for the quicker interpretation of results and increasing diagnostic accuracy [21,100,101,102], as well as creating convolutional neural networks that allow for a straight estimation of the distribution of materials, avoiding the need to perform the conventional material decomposition [103]. The development of algorithms to minimize noise and artifacts in VNCa images has yielded promising first results [104], and deep learning for photon-counting detector images has shown a greater performance in highlighting focal lesions [99].

Texture analysis offers quantitative information on aspects of images that are usually valued subjectively, such as heterogeneity or pattern repetitiveness. It has shown good preliminary results for the study of MM. Reinert et al., in 2020, proved a positive correlation between VNCa images between medullary infiltration in biopsy and advanced stages of the disease with medullary attenuation in textural analysis [105], as well as a good correlation between marrow image in pre- and post-treatment acquisitions and MM hematologic parameters [106]. Özgül et al. [107] demonstrated that machine learning-based CT texture analysis is a promising method for discriminating MM from osteolytic metastatic bone lesions.

FDG-PET/CT-based radiomics models implemented with machine learning algorithms can significantly improve the clinical prediction of progress and increase clinical benefits, providing prospects for clinical prognostic stratification for precision treatment, as well as new research areas [108]. 

Segmentation algorithms of MM lesions on WB-DWI have shown equally reproducible results when compared to the manual segmentation of radiologists. This semi-automatic segmentation method may aid in the accurate assessment of tumor burden and therefore provide insights into treatment response assessment [109]. Ekert et al. [110] studied MRI-based texture features, which proved significant in assessing clinical and hematological response in MM patients undergoing systemic treatment.

## 11. Conclusions

In MM, each whole-body imaging modality serves distinct roles in various disease scenarios. Therefore, clinicians must possess a comprehensive understanding of the applications, advantages, and disadvantages associated with each imaging method.

LDWBCT constitutes a precise screening tool for both bone and extramedullary findings. DECT is a promising technique with the potential to overcome some of the limitations of conventional CT, such as the detection of bone marrow infiltration without osteolysis, and to monitor treatment response in lytic lesions. 

MRI has the highest sensitivity in detecting lesions and spine complications, while FDG-PET/CT has a major impact on prognosis, and it is considered the preferred imaging technique for monitoring the metabolic response to therapy. WB-DWI can aid in differentiating benign entities, particularly hematopoietic bone marrow, and assess tissue cellularity with high sensitivity, challenging the dominance of FDG-PET/CT in evaluating treatment response. 

Both the adoption and implementation of structured reporting represent crucial strategies for improving communication between clinicians. Standardized terminology used to convey diagnostic certainty enhances the quality of patient care. Promising advances are photon-counting CT and artificial intelligence, both of which have the potential to revolutionize the radiological management of MM.

Imaging has gained unprecedented importance in assessing the extent of MM, establishing prognosis, detecting complications, and evaluating treatment response. Its significance is expected to further increase in the years ahead.

## Figures and Tables

**Table 1:** Advantages and disadvantages of all the different imaging modalities in patients with MM.

	LDWBCT	DECT	WBMRI	FDG-PET/CT	PET/MRI
Advantages	- Widely available - Short acquisition time and comfortable positioning - Allows the detection of lytic lesions and extraosseous disease - Good sensitivity, high specificity, and positive predictive value in presence of lytic lesions - Good interobserver correlation - Guide for biopsy and RT planning - Asses spinal stability	- Same advantages as conventional WBCT - Potential qualitative and quantitative assessment of bone marrow composition - Potential use in monitoring response to treatment and disease activity	- No radiation - Relatively available - Depicts lytic lesions and extraosseous disease and detects early non-lytic infiltration with the highest sensitivity - Prognostic significance of number of focal lesions and diffuse pattern - Extraosseous assessment - Superior assessment of spinal cord/nerve compression and secondary damage - Assessment of therapy response using DWI	- CT component allows the detection of lytic lesions and extraosseous disease - Prognostic significance of focal lesion and SUVmax value - Assessment of treatment response in MM (greatest advantage)	- Relatively new technique that combines the strengths of PET and MRI
Disadvantages	- Radiation exposure (relatively low) - Low negative predictive value in absence of lytic lesions - Unable to distinguish tumor infiltration from red marrow and active from inactive treated lesions, and equivocal for patchy osteoporosis - Limited assessment of spinal cord/radicular compression and soft tissue masses	- Radiation exposure (relatively low) - Not validated or included in clinical guidelines - Possible variability depending on vendor and postprocessing software - Limited availability - Unable to distinguish tumor infiltration from red marrow	- High cost - Not widely implemented - Long examination time - Not suitable for patients with pain or claustrophobia	- High cost - Limited availability - Limited assessment of spinal cord/radicular compression - False-negative FDG-PET/CT results in patients with lack of hexokinase (at least 10%). [11C] or [18F]-choline and [11C]-acetate are needed	- Very limited availability - Extremely high cost - Not validated or included in clinical guidelines - Depending on the institution, either PET-MRI or FDG-PET/CT may be chosen (in USA, not in Europe)
	- No need of iv contrast	- No need for iv contrast	- No need of iv contrast (optional)	- No need of iv contrast (optional)	- No need of iv contrast (optional)

LDWBCT: low-dose whole-body computed tomography; DECT: dual-energy CT; WBCT: whole-body computed tomography; WBMRI: whole body magnetic resonance imaging; DWI: diffusion-weighted imaging; FDG-PET/CT: 18F-fluorodeoxyglucose positron emission tomography/computed tomography; PET/MRI: positron emission tomography/magnetic resonance imaging.

## Data Availability

No new data were created or analyzed in this study. Data sharing is not applicable to this article.

## Supplementary Materials

The following supporting information can be downloaded at: https://www.mdpi.com/article/10.3390/jcm13010264/s1, Figure S1: Dynamic contrast-enhanced MR curve types; Figure S2: Novel scoring system to predict the risk of pathologic fracture in patients with multiple myeloma-related bone lesions; Figure S3: Spinal Instability Neoplastic Scale; Figure S4: Epidural Spinal Cord Compression Scale.
