# Supplementary material for: Imaging of Multiple Myeloma: Present and Future
Source: J Clin Med. 2024 Jan 2;13(1):264. doi: 10.3390/jcm13010264 (PMC10780302; doi:10.3390/jcm13010264)
Supplement: Supplementary file 1 [file jcm-13-00264-s001.zip › jcm-2697276-supplementary.pdf]

Supplementary Files:

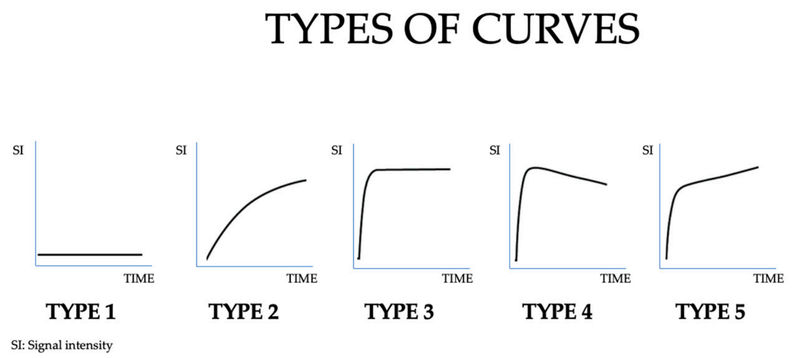

Figure S1. Dynamic contrast-enhanced MR curve types.

**Category**	**Point value**
Lesion latency [a]	
< 6 months	0
6 months to 1 year	2
> 1 year	4
Lesion size (cm2)	
< 5	0
≥5	2
Lesion location	
Diaphysis or epiphysis	0
Metaphysis	3
Pain[b]	
No	0
Yes	2

[a] From diagnosis of multiple myeloma to identification of the lesion of interest.
[b] Functional means worsened by activity or loading of the bone.

Figure S2. Novel scoring system to predict the risk of pathologic fracture in patients with multiple myeloma-related bone lesions.

ELEMENT OF SINS	SCORE
LOCATION	
Junctional (occiput-C2, C7–T2, T11–L1, L5–S1)	3
Mobile spine (C3–C6, L2–L4)	2
Semi-rigid (T3–T10)	1
Rigid (S2–S5)	0
PAIN RELIEF WITH RECUMBENCY AND/OR PAIN WITH MOVEMENT/LOADING OF THE SPINE	
Yes	3
No (occasional but not mechanical)	1
Pain free lesion	0
BONE LESION	
Lytic 2	2
Mixed (lytic/blastic)	1
Blastic	0
RADIOGRAPHIC SPINAL ALIGNMENT	
Subluxation/translation present	4
De novo deformity (kyphosis/scoliosis)	2
Normal alignment	0
VERTEBRAL BODY COLLAPSE	
50% collapse	3
50% collapse	2
No collapse with 50% body involved	1
None of the above	0
POSTEROLATERAL INVOLVEMENT OF THE SPINAL ELEMENTS (facet, pedicle or costovertebral joint fracture or replacement with tumor)	
Bilateral	3
Unilateral	1
None of the above	0

Figure S3. Spinal Instability Neoplastic Scale.

**Grade 0:**	Osseous disease only
**Grade 1a**:	Epidural involvement without thecal sac deformation
**Grade 1b**:	Thecal sac deformation without cord contact
**Grade 1c**:	Thecal sac deformation with cord contact
**Grade 2:**	Spinal cord/cauda equina/radicular compression with fluid (even minimal) around the neural tissue
**Grade 3:**	Spinal cord/cauda equina or radicular compression, with no visible CSF around the neural tissue

Figure S4. Epidural Spinal Cord Compression Scale
